# Facility type and primary care performance in sub-district health promotion hospitals in Northern Thailand

**DOI:** 10.1371/journal.pone.0174055

**Published:** 2017-03-24

**Authors:** Nithra Kitreerawutiwong, Sue Jordan, David Hughes

**Affiliations:** 1 Faculty of Public Health, Naresuan University, Phitsanulok, Thailand; 2 College of Human & Health Sciences, Swansea University, Swansea, Wales, United Kingdom; TNO, NETHERLANDS

## Abstract

**Background:**

Poor and middle-income Thai people rely heavily on primary care health services. These are staffed by a range of professionals. However, it is unknown whether the performance of primary care varies according to the staffing and organization of local service delivery units. Tambon (sub-district) health promotion hospitals (THPHs) were introduced in 2009 to upgrade the services offered by the previous health centres, but were faced with continuing shortages of doctors and nurses. The Ministry of Public Health (MoPH) designated three categories of THPH, defined according to whether they were regularly staffed by a medical practitioner, a qualified nurse or non-clinical public health officers. This study aimed to compare the performance of primary care offered by the three different types of primary care facilities in one public health region of Northern Thailand (Public Health Region 2).

**Methods:**

A cross-sectional survey was undertaken in 2013. Data were collected on accessibility, continuity, comprehensiveness, co-ordination and community orientation of care from 825 patients attending 23 primary care facilities. These were selected to include the three officially-designated types of Tambon (sub-district) health promotion hospitals (THPHs) led by medical, nursing or public health personnel. Survey scores were compared in unadjusted and adjusted analyses.

**Results:**

THPHs staffed only by public health officers achieved the highest performance score (Mean = 85.14, SD. = 7.30), followed by THPHs staffed by qualified nurses (Mean = 82.86, SD. = 7.06). THPHs staffed by a doctor on rotation returned the lowest scores (Mean = 81.63, SD. = 7.22).

**Conclusions:**

Differences in overall scores resulted mainly from differences in reported accessibility, continuity, and comprehensiveness of care, rather than staff skill-mix *per se*. Policy on quality improvement should therefore focus on improving performance in these areas.

## Introduction

Since the 1970s there has been widespread consensus that primary care offers the best prospect of providing health for all, while maximizing equity and efficiency [1–3]. Primary care offers access to treatments for all population subgroups at lower cost compared to specialist secondary care [4] and achieves greater service efficiency with better health outcomes, including lower standardized mortality rates, lower rate of premature death, higher infant birth weight, life expectancy, public satisfaction and cost containment [2, 5–7]. For more than three decades the WHO has encouraged countries around the world to strengthen their health care systems through the values and principles of primary care-led healthcare [8].

The Alma Alta Declaration [9] identifies primary care as “the first level of contact of individuals, the family and the community with the national health system, bringing health care as close as possible to where people live and work, and constituting the first element of a continuing health care process” (p.16). The US Institute of Medicine [10] defines the function of primary care as “the provision of integrated, accessible health care services by clinicians who are accountable for addressing a large majority of personal health care needs, developing a sustained partnership with patients, and practicing in the context of family and community” (p.31).

Primary care has been central to Thai healthcare reform for several decades. From the 1970s onwards the Ministry of Public Health channeled resources to build a strong ‘district health care’ system. An average district with a population of about 50,000 people is served by a community hospital with 30–120 beds and 100–300 staff, including doctors, nurses, dentists, and pharmacists, and by 10–15 sub-district health centres, each with 3–5 paramedical staff. The local health network of hospital and health centres is able to refer more complex cases to provincial and tertiary hospitals within the Minstry of Public Health system. Although Thailand has been training more doctors and nurses since the 1970s, there has been a longstanding problem of shortages of professional staff in rural areas, where much of the burden of care fell on public health officers and village health volunteers. Mandatory rural service for doctors and nurses, and more recently dentists and pharmacists, has reduced but not eliminated this problem of uneven workforce distribution [11].

Thailand’s 2001 universal health coverage (UHC) reforms aimed to strengthen primary care by giving local primary care networks greater control of financial resources, and further developing “close to the home, close to the heart” community facilities [12]. The Thai system attempts to integrate medical and public health services so that health promotion and disease prevention run alongside curative care. In the original 2001 UHC model the district health networks received capitation-based funding for their population that covered services provided within the network and also the costs of referral to secondary care, thus empowering local managers [13]. A typical district health system consists of a contracted unit for primary care (CUP) based on a district hospital, which holds the budget, and its network of associated health centres. The CUP supports its local network by overseeing the organization of finance and human resources, providing local diagnostic and treatment facilities (usually via the district hospital), and where necessary arranging upward referrals [14]. The Thai primary care system puts strong emphasis on coordination between professionals and local communities, so as to develop self-care knowledge relevant to illness [15].

Although many international observers regard the Thai UHC reforms as a major success story [16, 17], attempts to improve community health services made only slow gains. The move to divert a greater share of funding into primary care was reversed in the 2000s as hospital budgets came under pressure [13]. The policy to develop health centres into more capable primary care units (PCUs) was hampered by shortages of doctors and nurses [18]. Generally these facilities are staffed by public health officers who have degree-level qualifications in health promotion and disease prevention, but are also called upon to offer basic treatments. In 2009, in an attempt to rectify widely acknowledged weaknesses in local services, these community facilities were reorganized to form sub-district (or Tambon) health promotion hospitals (THPHs), based on the existing health centre facilities.

The THPHs were not intended to be inpatient facilities with staffed beds, but rather a type of polyclinic that could serve as a “node” for delivering preventive medicine and health promotion and treatment for minor trauma or non-serious illness. The THPHs also provide access to pharmaceuticals from the national essential drugs list [19]. The THPH policy involved rotating medical and nursing staff from district hospitals to work sessions in the THPHs and provide diagnostic and curative services at sub-district level. A typical sub-district (Tambon) might comprise two or three villages, and a thousand to five thousand people. The THPH initiative involved re-furbishing the health centre estate, upgrading, equipment, and providing ambulance transport so as to encourage patients to seek close-to-home care rather than visiting the district hospital.

The THPH initiative represents an attempt to raise the quality of primary care services by improving integration at local level, especially by utilizing professional staff from the district hospital in the THPHs [20]. The three occupational groups who play key roles in the Thai rural health system are doctors, nurses, and public health officers. The professional expertise and authority of doctors would seem to place them centre stage, and many commentators portray doctor shortages as the main source of quality problems in rural primary care. However, nurses have become important middle-level care providers who are able to fill in gaps when doctors are unavailable, with substitution being one way of controlling costs [21, 22]. Public health officers are the largest staff group, and in theory concentrate on health promotion/disease prevention, while lacking formal training to provide anything beyond simple curative care [14].

Under the 2009 THPH policy the MoPH set targets for the number of health care personnel in each primary care facility [20]. However, few THPHs were able to achieve the specified staff complement and implement multidisciplinary teamwork. Prior to the THPH initiative, only 2% of district hospital-based CUPs were able to allocate a doctor to a health centre on a rotation basis [23], while around 70% of CUPs attached a registered nurse or nurse practitioner [24]. Data collected in 2010 by the MoPH Office for Tambon Health Promotion Hospitals showed only 82 doctors working in THPHs across the nation [25], reflecting the inequitable distribution of the medical workforce. The deficit is obvious given that 35,789 doctors serve a Thai population of over 62.6 million [26]. Although the majority of Thais reside in rural areas there is an over-concentration of the medical and nursing workforces in urban centres [14].

As a practical response to these problems, the Ministry of Public Health authorized the designation of three types of THPH: (1) main node THPHs with a doctor on rotation (THPH DR), (2) THPHs with no doctor but a nurse practitioner or registered nurse (THPH NU) and (3) THPHs staffed only by public health officers (THPH PH). THPHs DR is mainly found in city (or Muang) districts, while the THPHs NU and THPHs PH are common in rural areas.

This study investigated how far performance differs in these three THPH types, measuring primary care performance using a number of pre-defined dimensions. Information on performance differences should be useful to policy makers deciding how to allocate resources to support primary care, to health practitioners seeking to improve primary care services, and for effective primary care education.

The Bureau of Primary Care Development Coordination [15] has suggested that Thai primary care has five important dimensions. First it should be accessible, so that service users can obtain initial care for most health needs from their registered primary care facility. Second the care package must be comprehensive in that it includes all necessary services, including health promotion, disease prevention, and curative, rehabilitative and supportive care for long-term conditions, provided both in the clinic and the home. Thirdly, services need to be coordinated so that local providers are able to refer service users to other specialized facilities and monitor progress on an ongoing basis. Fourth, the system needs to achieve continuity of care, with providers maintaining accessible medical records and meeting patients’ health needs over time. Finally care should have a community orientation, so that providers manage health problems at the population as well as the individual level, and the community has some influence on how primary care services are delivered. These five dimensions have been adopted for use in several past studies examining primary care [27–29], but to date no attempt has been made to utilize them to evaluate the performance of primary care facilities in Thailand.

A recent Korean study, which illustrates this general approach, found that health cooperatives scored more highly than public health centre clinics on a similar set of dimensions [30]. A Hong Kong study using much the same approach reported that private general practice clinics achieved higher scores than general outpatient clinics, which however provided care for an older, poorer and less educated population with more chronic illness [31]. We suggest that the dimensions of accessibility, comprehensiveness, coordination, continuity and community orientation are associated with quality of service performance [32], patient satisfaction [33], and health system effectiveness, defined as improving health outcomes [34], and can therefore be combined into a useful tool for measuring the performance of primary care [32].

## Methods

This study was a cross-sectional survey of patients who received services at selected THPHs from January to June 2013. It concentrated on patient perspectives, reflecting the growing emphasis in many healthcare systems on public engagement and patients’ rights to express views on service quality [35]. The study was approved by the Naresuan University Research Ethics Committee and permission for research access was obtained from the Ministry of Public Health and the THPHs concerned.

### Setting

Public health Region (Keet) 2, which covers the five lower northern provinces of Phitsanulok, Petchboon, Sukothai, Utaradit, and Tak, was purposively selected on the basis that it covers both urban and rural populations and thus contains a good mix of the three facility types of interest in the study.

### Sample and recruitment

The study aimed to investigate patients’ perceptions of service performance in THPHs in all five provinces of the health region across all age groups and including beneficiaries of all three public health insurance schemes. This involved a multi-stage process of first sampling districts, and then sampling THPHs and patients attending each facility. An urban (municipal) district and a rural district were purposely selected in each province, taking account of registered UHC beneficiaries so as to get a spread of district sizes. This yielded a total of 10 districts in the study, each with a community hospital and its network of THPHs. As far as possible the study then purposely selected one THPH of each of the three types in each district, including a range of facility sizes. However, none of the five urban districts operated THPH PHs, and one of the rural districts lacked a THPH DR. This meant that 23 THPHs were included, comprising 9 THPHs using doctor rotation, 10 THPHs led by nurses, and 4 THPHs staffed solely by public health officers. These ratios broadly reflected the pattern across the region where this last type was found only in rural districts. All the facilities approached granted research access so that the original sampling plan was preserved.

Having gained access to the 23 THPHs, a sample of patients was then recruited in each facility (S1 File). The sample size was estimated based on a mean for infinite population [36]. A previous study in Taiwan, in a primary care setting within a universal coverage system with some similarities to that of Thailand, reported a standard deviation of 3.4 [37]. Thus taking a margin of error of ±0.25, the sample size was calculated at 711. Anticipating a ~20% nonresponse rate [38], we distributed 851 questionnaires. We aimed to recruit equal numbers of respondents in each district and type of facility. Based on these considerations, the researchers set out to recruit 37 patients in each of the 23 primary care facilities.

This was a convenience sample selected by recruiting successively-arriving eligible patients until the required number was achieved. The lead researcher and 4 assistants, who were trained in participant recruitment and the details of this questionnaire, approached all patients (or accompanying adults responsible for patients aged 15–17) in the waiting area. Younger children and unaccompanied minors aged 15–17 years were excluded. Each potential participant was given an explanation of the research and a chance to ask questions before deciding whether to participate. They were then asked if they met the requirements for inclusion. The inclusion criteria were that the patient was aged 15 years (the lower threshold for Thai ‘adult services’) and over, and did not have a serious acute condition, cognitive impairment or known psychiatric disorder. This information was gained from the patients or responsible adults and verified by checking patient records. Data collection was managed so as to cover both morning and afternoon sessions over a 7 day period to minimize selection bias.

Following the health care contact, patients aged 18 and over were asked to complete a questionnaire on their own behalf, while responsible adults were asked to complete the questionnaire for patients under 18. Most completed the instrument themselves in meeting rooms in the facilities, in some cases asking for assistance from the researchers who were on hand to help. A minority with literacy problems or sight impairment gave verbal answers that were written in by the researcher. The researchers ceased recruitment once it was judged that 37 completed questionnaires from each of the 23 sites (total 851) had been obtained. However, because 26 returned questionnaires were less than 80% complete and were therefore excluded, only 825 were usable for analysis.

### Research ethics

All adult research participants gave informed consent. The researchers explained the purpose of the study to each adult patient or responsible adult helper, who was also given an information sheet and the opportunity to ask questions before deciding whether to participate. Assurances of anonymity and confidentiality were given verbally and on the information sheet. Willing participants signed a consent form before questionnaires were completed and passed to/ retained by the research team. It was assumed that it was acceptable for adult helpers to give consent on behalf of minors aged 15–17. Agreement to research access was obtained in writing from the Ministry of Public Health, the 5 provincial health offices, the 10 district health offices and the 23 THPHs selected. The study and its proposed ethical safeguards and consent procedure were approved by the Naresuan University Research Ethics Committee (reference HE 56-Ex2b-0004).

### The questionnaire

This study adopted a conceptual framework of primary care attributes used by Kitreerawutiwong and associates [27] (S2 File). The primary care attributes questionnaire (PCAQ) examines patients’ experience of primary care services, rather than their satisfaction with care, and consists of 5 domains: (1) accessibility, (2) continuity, (3) comprehensiveness, (4) coordination, and (5) community orientation, (25 questions in total). The questionnaire uses 5 Likert scale items that allow responses on a 5-point scale, with 1 signifying strong disagreement and 5 strong agreement. The total score for each domain is calculated by summing responses, with a possible range of 25–125. The score was transformed into a scale of 0–100 to facilitate analysis, with higher scores indicating more favorable performance. The raw scores for each domain were totaled and converted into a scale of 0–100 points using the formula recommended in Nanyonjo et al's primary care assessment survey (the transformed scale score = [(mean item value−lowest possible value) ÷ possible item value range]×100) [39]. Demographic data (e.g. sex, age, marital status, education, and income), membership of health insurance scheme, and number of visits to a primary care facility in the last year were also collected.

The PCAQ was validated using an item content validity index (I-CVI) to assess the degree of agreement among five experts. These experts included one medical doctor who worked at the provincial health office, two nurse practitioners who worked in primary care services at district and sub-district level and two academics specializing in primary care and healthcare systems. There was a high degree of agreement among the experts, with a resultant I-CVI score of 1. After the instrument had been validated by the panel, it was subjected to further pilot testing to assess reliability with 30 patients in Nakhonsawan Province. Reliability was measured in terms of internal consistency using Cronbach’s alpha coefficient. The overall Cronbach’s (α) of 0.88 with five dimensions ranging from 0.77 to 0.84, and this was judged to be satisfactory.

## Data analysis

The data were entered into SPSS version 19 for analysis [40]. Primary care performance scores were calculated for the five domains or quality attributes, and distributions were checked. Descriptive statistics—frequency, mean and standard deviation—were calculated to assess the level of primary care performance by facility type. The chi-square test was used to test for differences in demographic characteristics of the three samples relating to the three facility types. Scores for each facility type were compared using unadjusted Analysis of Variance (ANOVA). Where ANOVA suggested differences, multiple comparisons were undertaken. The Least Significant Difference (LSD) test was utilized to compare differences between the mean values of the 3 facilities to examine the differences in the scores for accessibility, continuity of care, and comprehensiveness among the three type facilities. Analysis of covariance was undertaken to adjust for age, marital status, and education. Selection of variables was based on univariate testing. The level for statistical significance was set at 2 sided alpha< 0.05.

We investigated a range of outcome (or dependent) variables; however, we made no *a priori* assumptions that these would be related. Given that our analysis involves several dependent variables, the feasibility of a multivariate analysis of variance (MANOVA) was considered. Unlike ANOVA, MANOVA examines covariance between outcome variables to test the significance of mean differences, and requires additional assumptions regarding the data, such as the absence of multivariate outliers, multivariate normality, linear relationship between the dependent variables, low multicollinearity between dependent variables and homogeneity of variance-covariance matrices. Unfortunately, in our data some of these assumptions were not met. Consequently, and given that our groups were of unequal size, we concluded that a MANOVA could not be employed.

## Results

883 patients were approached, and 32 declined to participate due to such factors as family commitments or travel demands and were not given questionnaires. 851 questionnaires were distributed to those consenting to take part. 26 returned questionnaires failed to meet the discard threshold of being 80% complete and so were excluded, thus leaving 825 questionnaires available for analysis. Thus the response rate was 96.38% (851/883) and the usable response rate was 93.43% (825/883).

A total of 310 (37.58%) questionnaires were completed at medically staffed THPHs (THPH DR), 385 (46.67%) at those staffed by nurses (THPH NU), and 130 (15.76%) at those staffed by public health officers (THPH PH). A majority of respondents were female (68.61%) with respondents aged from 18 to 85 years (mean = 45.58 years, SD. 14.45). Most (73.21%) were married. A majority (54.30%) had completed at least primary school education. Agriculture was the primary occupation of nearly half (48.73%) of the respondents. The median income was 5,000 baht per month (approximately GBP £100 or US $150) compared with a reported average of 7,500 baht/month for 5 provinces in 2013 [32]. Most respondents (84.73%) were insured under the public Universal Coverage Scheme (UCS), with the Social Security Scheme (for those working in larger companies) and the Civil Service Medical Benefits Scheme (for a wide range of public servants including teachers and health workers) each accounting for just over 7% of respondents. Just over 1% paid out of pocket (as for example, may be required for UCS patients away from their home area). Approximately two thirds (62.79%) had visited a primary care facility at least 3 times in the last year. The distribution of age, marital status, education, occupation, income and insurance differed significantly between different facility types, but sex and number of visits did not (Table 1).

**Table 1:** Characteristics among clients in different type of primary care facilities (n = 825) [Figures are n (%) except as indicated].

Characteristic	THPH DR^a^	THPH NU^b^	THPH PH^c^	Total	df	p-value^a^
	(n = 310)	(n = 385)	(n = 130)	(n = 825)		
**Age (year), figures are means (SD)**	48.34(14.93)	46.02(13.57)	37.88(12.91)	45.62(14.41)		<0.01^†^
**Range (min,max)**	(15,85)	(15,82)	(17,76)	(15,85)		
**Sex**						
Male	98(31.61)	118(30.65)	43(33.08)	259(31.39)	2	0.87
Female	212(68.39)	267(69.35)	87(66.92)	566(68.61)		
**Marital status**						
Single	47(15.16)	44(11.43)	15(11.54)	106(12.85)	4	0.04^†^
Married	211(68.06)	288(74.81)	105(80.77)	604(73.21)		
Divorced/Windowed/Separated	52(16.77)	53(13.77)	10(7.69)	115(13.94)		
**Education**						
Primary	166(53.55)	222(57.66)	60(46.15)	448(54.30)	4	<0.01^†^
Secondary	90(29.03)	119(30.91)	60(46.15)	269(32.61)		
Above secondary	54(17.42)	44(11.43)	10(7.69)	108(13.09)		
**Occupation**					10	
Agriculture	115(37.10)	182(47.27)	105(80.77)	402(48.73)		<0.01^†^
Merchant	33(10.65)	52(13.51)	3(2.31)	88(10.67)		
Other private-sector employee	80(25.81)	87(22.60)	16(12.31)	183(22.18)		
Government worker	17(5.48)	14(3.64)	0	31(3.76)		
Housewife	44(14.19)	30(7.79)	2(1.54)	76(9.21)		
Retired or unemployed elders	21(6.77)	20(5.19)	4(3.08)	45(5.45)		
**Income (Baht**^‡^**/month)**						
≤ 7,500	165(53.23)	259(67.45)	121(93.08)	545(66.14)	2	<0.01^†^
> 7,501	145(46.77)	125(32.55)	9(6.92)	279(33.86)		
Mdn (Min, max) = 5,000 (500,100000)						
**Insurance**						
Universal Coverage Scheme	248(80.00)	327(84.94)	124(95.39)	699(84.73)	6	<0.01^†^
Civil Servant Medical Benefits Scheme	32(10.32)	25(6.49)	1(0.77)	58(7.03)		
Social Security Schem	25(8.06)	29(7.53)	5(3.85)	59(7.15)		
Private/ self funding	5(1.55)	4(1.04)	0	9(1.09)		
**Frequency of visiting primary care facilities (No.) in the last year**					2	
≤ 3 times per year	189(60.96)	247(64.16)	82(63.08)	518(62.79)		0.69
> 3 times per year	121(39.04)	138(35.84)	48(36.92)	307(37.21)		

^†^ Significant differences of ANOVA or chi square test (p value < 0.05)

^‡^ 1 US$ = approximately 30.3 Baht

^a^ THPH DR is defined as Tambon Health Promotion Hospital with a doctor working on a rotation basis

^b^ THPH NU is defined as Tambon Health Promotion Hospital with registered nurse or nurse practitioner

^c^ THPH PH is defined as Tambon Health Promotion Hospital with public health officer.

Table 2 shows the results relevant to performance of the three facility types, with and without adjustment for age, marital status and education. For most domains, PH had the highest scores and DR the lowest. The exception was co-ordination, where nurses had the lowest scores. In unadjusted comparisons there were no significant differences in continuity of care, coordination, and community orientation. The average accessibility score was highest (mean = 84.67, SD. = 6.87) in the facilities staffed by public health officers (THPH PH), followed by the THPH NU (mean = 82.84, SD. = 7.24). In unadjusted analyses, there were statistically significant differences in scores for accessibility and comprehensiveness, and total scores, with the THPH PHs receiving the highest scores. After controlling for age, marital status and education, there were no significant differences in coordination and community orientation differences in accessibility, comprehensiveness and total scores remained, and continuity of care became significant.

**Table 2:** Care performance scores in three types of primary care facilities (n = 825).

			Type of primary care facilities			Unadjusted analysis^§^		Adjusted analysis^#^	
Primary care attributes	No. of items	Range of scores	THPH DR^a^ Mean (SD)	THPH NU^b^ Mean (SD)	THPH PH^c^ Mean (SD)	F_(2,822)_	P^†^-value	F_(2,822)_	P^†^-value
Accessibility	7	7–35	82.19(8.96)	83.20(8.77)	87.10(12.03)	12.64	<0.001^†^	18.56	<0.001^†^
Continuity of care	4	4–25	83.71(10.39)	84.81(9.31)	86.08(9.30)	2.88	0.06	8.29	0.001^†^
Comprehensiveness	3	3–15	82.68(8.46)	83.84(8.95)	85.36(7.71)	4.62	0.01^†^	5.66	0.004^†^
Coordination^‡^	7	7–35	83.62(12.72)	79.39(8.47)	81.43(5.74)	2.04	0.13	2.07	0.13
Community orientation	4	4–25	79.05(12.76)	79.68(12.05)	80.42(11.76)	0.61	0.55	0.51	0.60
Total score	25	25–125	81.85(7.50)	82.84(7.24)	84.67(6.87)	6.94	0.001^†^	10.67	0.001^†^

^†^ Statistical probabilities calculated using ANOVA

^‡^ n = 114, only included patients who were referred to another health care or non-health sector facility

^§^ statistical probabilities calculated using analysis of covariance, adjusting for age, marital status, and education for unadjusted analysis

^#^ statistical probabilities calculated using analysis of covariance, adjusting for age, marital status, and education for adjusted analysis

^a^THPH DR is defined as Tambon Health Promotion Hospital with a doctor working on a rotation basis

^b^THPH NU is defined as Tambon Health Promotion Hospital with registered nurse or nurse practitioner

^c^THPH PH is defined as Tambon Health Promotion Hospital with public health officer.

LSD confirmed that facilities staffed by public health officers (THPH PH) indeed scored more highly than other facility types on these three dimensions (Table 3).

**Table 3:** Multiple comparisons: analysis of accessibility, continuity, comprehensiveness, coordination, community orientation and total scores.

Attributes	Type of primary care facility											
	THPH DR^a^ & THPH NU^b^				THPH DR^a^ & THPH PH^c^				THPH NU^b^ & THPH PH^c^			
	Mean difference (SE)	95% CI		p	Mean difference (SE)	95% CI		p	Mean difference (SE)	95% CI		p
Accessibility	-1.23(0.72)	-2.64	1.83	0.88	-6.87(1.10)	-9.03	-4.70	<0.001*^†^	-5.64(1.07)	-7.74	-3.54	<0.001*^†^
Continuity	-1.47 (0.73)	-2.90	-0.03	0.05^†^	-4.58(1.12)	-6.78	-2.38	<0.001^†^	-3.11(1.09)	-5.25	-0.98	0.004^†^
Comprehensiveness	-1.29 (0.66)	-2.59	0.09	0.05	-3.05 (1.02)	-5.05	-1.06	0.003^†^	-1.76 (0.98)	-3.70	0.17	0.074
Coordination	3.96(2.15)	-0.31	8.23	0.07	1.98(2.94)	-3.85	7.81	0.503	-1.99(2.96)	-7.84	3.87	0.503
Community orientation	-0.66(0.95)	-2.52	1.20	0.49	-0.29(1.45)	-3.14	2.56	0.841	0.37(1.41)	-2.39	3.13	0.793
Total	-1.17(0.57)	-2.26	-0.08	0.04	-3.64(0.85)	-5.31	-1.97	<0.001^†^	-2.47(0.83)	-4.10	-0.85	0.003^†^

*^†^Significant differences of accessibility in THPH DR vs. THPH PH and THPH NU vs. THPH PH in multiple comparisons using LSD (*p* < 0.05).

^†^ Significant differences of continuity of care, comprehensiveness, and total score in THPH DR vs. THPH NU, THPH DR vs. THPH PH and THPH NU vs. THPH PH in multiple comparisons using LSD (*p* < 0.05).

^**a**^THPH DR is defined as Tambon Health Promotion Hospital with a doctor working on a rotation basis,

^**b**^THPH NU is defined as Tambon Health Promotion Hospital with registered nurse or nurse practitioner,

^**c**^THPH PH is defined as Tambon Health Promotion Hospital with public health officer.

## Discussion

The attributes used here to measure primary care performance have been utilised in previous studies [30, 41, 42]. Other research has assessed performance by examining the organisation of primary care facilities [30, 31, 43] or insurance type [44]. This study set out to examine the impact of different staffing arrangements—an issue directly relevant to Thai government policies on workforce planning, teamwork and skill-mix [45]. Currently the MoPH must decide spending priorities, and to what extent strengthening the professional workforce should be prioritized above spending on infrastructure, health technology and medicines. The Ministry’s existing commitment to interdisciplinary primary care and team working raises questions about the costs and benefits of changing the professional skill-mix. Counter-intuitively, the results of this study showed that facilities staffed only by public health officers achieved the highest service user scores for accessibility, comprehensiveness and continuity of care in the adult patients surveyed. Surprisingly, the facilities staffed by doctors received the lowest scores, also falling behind nurse-led THPHs. The study did not investigate the reasons for the paradoxical finding that the most highly qualified professionals generate the lowest performance scores, but, based on our experience, we may speculate that the explanation lies in several organizational and contextual factors affecting the different types of THPHs.

One reason why the THPH DRs scored lowest may be that medical cover in these facilities is not continuous. In many of these THPHs doctors attend on a rotation basis only for one or two clinic sessions of 3–6 hours per month. Doctors may also be moved frequently between different CUP facilities with negative consequences for continuity of care. Since 1986 the government has attempted to improve equity in the distribution of the health workforce by enforcing compulsory contracts with medical students so that they must perform three years of public service after graduation or face high fines [46]. The physicians staffing THPHs are likely to be relatively newly-qualified professionals with no postgraduate training in primary care and limited experience of family practice. Jaturapatporn [47] found that family physicians with postgraduate qualifications had the highest General Practitioner Assessment Questionnaire (GPAQ) scores for many dimensions of family practice skills such as continuity of care, communication and enablement, when compared with general physicians and residents. Hospital physicians rotated to the THPHs are often strong in a particular clinical specialty but unfamiliar with the full range of primary care activities, and so cannot deliver the type of care expected. They provide “OPD like”, first line care, for which they are trained, but which may be deficient from the standpoint of service users expecting continuity of care and a patient-centred approach [48].

One strand of the Thai UHC reforms attempted to improve community facilities under the policy slogan of “*glai-baan glai-jai*” (“close to the home, close to the heart”) care. THPHs continued this policy by maintaining sites in rural locations convenient to local populations [49], and these rural THPHs were the ones likely to be staffed by public health officers (80.7% of sampled patients in THPH PH were employed in agriculture compared with lower figures for the other two types). The fact that THPH PHs tend to be away from urban centres probably explains why they rated higher for accessibility than the other two types. This suggests that location was indeed important to rural dwellers, especially when coupled with convenient transportation and short traveling time.

The high ratings for continuity of care from THPH PH patients suggest that they see professional carers on a regular basis, and that the relationship between service provider and patient extends beyond specific episodes of illness or disease. Public health officers are rarely rotated between facilities as happens with doctors, and are therefore better able to offer continuity of care over time.

Comprehensiveness scores were assessed in relation to health promotion, counseling, and disease surveillance. The lower scores achieved by THPH DRs may reflect their role as nodes of primary care which will attract patients needing curative services from beyond the immediate area, and push doctors towards spending most of their time on clinical treatments and screening. As a result these facilities allocate less time to activities like health promotion and counseling and achieve lower all-round scores.

The message of this study is not that increasing numbers of doctors and nurses in rural areas is a bad thing, but rather that improved physician and nurse staffing needs to be introduced in a way that preserves positive characteristics of the local health system such as accessibility, comprehensiveness, and continuity of care. Past experience suggests that shortages of professional staff in rural clinics lead patients to present directly at district and provincial hospitals and overburden services there [50]. Moreover if implemented correctly, Thailand’s existing policies on multi-disciplinary working will bring about a division of labour that gives valuable roles to doctors, nurses and public health officers. The danger is that the drive to ensure access to professional staff in the face of workforce shortages will lead to a re-focusing on the hospital as the main treatment site. If patients are required to attend OPD-like facilities within district hospitals to see physicians, or to travel increased distances to attend larger ‘node’ THPH DRs, they may believe that the quality of services has fallen. The present study suggests that people value “close to the home” care, provided by professionals whom they know, and offering a wide range of curative, preventative and supportive services, and that access considerations may be just as important as the type of staff that they see.

Several limitations of this research need to be acknowledged. First, because this study covered only a single region, we must be cautious about generalizing the findings. We might hypothesize that they will apply to other areas with similar geographical and cultural characteristics, but further studies are needed to confirm this. Second, like other research designed to assess patients who received primary care services, this study did not provide data on those who did not use the local health care system [30, 42, 51], including persons dissatisfied with the service offered whose responses may have lowered performance scores. Thirdly, although primary health care in Thailand provides curative care, rehabilitation, preventive services, and health promotion, the latter three areas often involve projects or activities in the community, so that our study may relate mainly to patient perspectives on performance in curative care. Fourthly, the decision to sample facility types to reflect the proportions found in the region meant that the number of THPH PHs (which are less numerous than the other types) included was small, and this may raise issues of representativeness and generalizability beyond this region. Fifthly, patients were selected by convenience sampling, and were not necessarily representative of all service users. Against this, the high response rate (>90%) reduces the risk of volunteer bias. We cannot exclude social desirability response bias, but we have no reason to suspect that this would differentially affect any particular clinic type. We acknowledge the hazards of multiple testing, and present our pairwise comparisons as exploratory analyses, awaiting confirmation in further studies. Lastly, like the other studies of primary care performance (30,31,32,42,44,51) this research did not investigate technical quality, which would require a clinical outcome assessment component to be added to the study design. While studies like the present one that examine service users’ perceptions of performance are valuable, the authors accept that we need to build further to design studies that examine both primary care performance and clinical outcomes in relation to differences in the organization of local facilities.

## Conclusions

In summary, this study aimed to investigate differences in performance scores that might suggest which aspects of service delivery required improvement. The results have implications for both policy makers and practitioners. Service improvement may not be simply about increasing the numbers of professionally qualified staff, but also about the organization of services and close attention to the things that patients value. This study showed that patients who received primary care at THPHs staffed by public health officers gave the highest scores for accessibility and comprehensiveness of care. The positive rating of the THPH PH is particularly striking, especially given that their users are likely to be drawn from the most socially disadvantaged population groups, whose access to healthcare before the UHC reforms was quite limited. The study suggests that many THPHs still need to work to improve the accessibility, comprehensiveness and continuity of care, and that policy makers and practitioners should not lose sight of these considerations when they devise strategies to increase patient access to doctors and nurses.

## Supporting information

S1 File Annex on sampling: Information regarding selection of THPHs. (DOC)

S2 File Primary care assessment survey questionnaire. (DOC)
