# Supplementary material for: Facility type and primary care performance in sub-district health promotion hospitals in Northern Thailand
Source: PLoS One. 2017 Mar 24;12(3):e0174055. doi: 10.1371/journal.pone.0174055 (PMC5365112; doi:10.1371/journal.pone.0174055)
Supplement: S1 File — (DOC) [file pone.0174055.s001.doc]

**Annex on sampling: Information regarding selection of THPHs**

**Step 1**: Health region (Keet) 2 was selected for reasons of convenience (as the base of Naresuan University). Keet 2 contains five provinces, which were all included. These were: Phitsanulok, Uttradit, Petchaboon, Tak and Sukhothai.

**Step2**: Two districts were purposely select in each of the five provinces. In all provinces we selected the Muang (or provincial capital city) district as the representative of an urban area (there is only one Muang district in each province), and one Amphur (or mainly rural) district centring on a small town. Additionally the Amphur selection took account of size of registered UHC beneficiary population so as to include a spread of district sizes and number of THPHs per district

Our selections were:

Phitsanulok: Muang district (urban) = 22 THPHs + Phompiram district (rural) = 18 THPHs

Uttradit: Muang district (urban) = 21 THPHs + Nampad district (rural) = 10 THPHs

Petchaboon: Muang district (urban) = 22 THPHs + Lomsak district (rural) = 30 THPHs

Tak: Muang district (urban) = 21 THPHs + Maeramad district (rural) = 9 THPHs

Sukhothai: Muang district (urban) = 14 THPHs + Sawankalok district (rural) = 16 THPHs

**Step 3:** Purposive selection of one THPH of each of the three types in each study district. This was done in such a way as to give us a range of sizes of facility.

There are 183 THPHs in the selected districts in the five provinces. The number of urban areas vs rural areas was 100 vs 83. Facilities staffed only by public health officers - PH (THPH PH) – are the least common type because of government policy to allocate more nurses and doctors to rural THPHs.

| **Province** | **Muang** | | | **Rural** | | |
| --- | --- | --- | --- | --- | --- | --- |
|  | **THPH DR** | **THPH NU** | **THPH PH** | **THPH DR** | **THPH NU** | **THPH PH** |
| Phitsanulok | 1 | 1 | None | 1 | 1 | 1 |
| Uttradit | 1 | 1 | None | 1 | 1 | 1 |
| Petchaboon | 1 | 1 | None | 1 | 1 | None |
| Tak | 1 | 1 | None | None | 1 | 1 |
| Sukhothai | 1 | 1 | None | 1 | 1 | 1 |
| Total | 5 | 5 | None | 4 | 5 | 4 |
