# Supplementary material for: Facility type and primary care performance in sub-district health promotion hospitals in Northern Thailand
Source: PLoS One. 2017 Mar 24;12(3):e0174055. doi: 10.1371/journal.pone.0174055 (PMC5365112; doi:10.1371/journal.pone.0174055)
Supplement: S2 File — (DOC) [file pone.0174055.s002.doc]

**Primary care assessment survey questionnaire**

| Accessibility: the presence (or absence) of first point of contact, geographical, financial, organizational and structure barriers to received primary care services.   1. You have chosen this health-care facility as your first choice when you have a health problem. 2. This facility provides an out of hour services. 3. The steps of service provision from registration through getting medicine are not complicated. 4. You do not have any problems with expenditure when you receive health services at this facility. 5. Travelling from your home to this facility is convenient. 6. This facility can provide the services that suits your health needs. 7. The accommodations such as toilets, waiting area, and drinking water were adequate. |
| --- |
| Continuity of care: the presence (or absence) of continuity of services, informational continuity and relationship continuity.   1. The health officials regularly ask you about your health and livelihood when they visit you at home. 2. If you or your family members have health problems or conditions such as post-operation or post-delivery, elderly, disability, diabetes, hypertension or paralysis, a health official visits your family at home. 3. The providers recognize you and your family members. 4. The providers recognize and understand your community culture. |
| Comprehensiveness: To extent services to you and your family obtained the following services from the facility.  This facility provides services such as   1. Advice about appropriate exercise for you 2. Checking on and discussing the medications you are taking 3. Eradication of mosquito larvae and surveillance of communicable diseases such as diarrhea and leptospirosis |
| Coordination: Referral to other health providers and collaboration with non-health providers   1. The facility’s official has given you a referral letter to you when you need to see a medical specialist. 2. The facility’s official helps you with vehicle arrangements and making appointments with the referred hospital. 3. You think that the facility should have one official assigned to coordinate with the referred hospital when you need to receive such medical treatment. 4. You enjoyed the convenience provided through the coordination effort between this facility and the referred hospital. 5. When the clients have a socio-economic problem, the facility’s official will collaborate with other relevant organizations. 6. You want to have one official of the facility to help coordinate with other non health agencies such as provincial social welfare office, municipality or sub-district administrative organization, as needed. 7. You enjoyed the convenience provided through the coordination effort between this facility and other non-health agencies such as provincial social welfare office, municipality or sub-district administrative organization. |
| Community orientation: Providers manage health problems based on population approach and empower community members to provide inputs on how their primary care services can be improved   1. Primary care provider knows about the important health problems of their neighborhoods 2. Primary care providers get opinions and ideas from people that will help to provide better health care 3. You believe that the community should participate in the review and development of services at this facility. 4. You have participated with officials in the development of this facility. |
